# Meet the Editors‐in‐Chief

**DOI:** 10.1002/ansa.20190010

**Published:** 2020-03-24

**Authors:** Christoph Steinbeck, Paul Trevorrow

**Affiliations:** ^1^ Friedrich‐Schiller‐University Jena Germany; ^2^ Executive Journals Editor Wiley Chichester UK


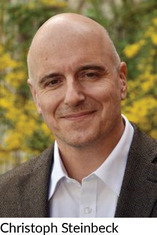


Christoph Steinbeck studied chemistry at the University of Bonn, where he received his diploma and doctoral degree at the Institute of Organic Chemistry. Focus of his Ph.D. thesis was the program LUCY for computer‐assisted structure elucidation. In 1996, he joined the group of Prof. Clemens Richert at Tufts University in Boston, MA, where he worked in the area of biomolecular NMR on the 3D structure elucidation of peptide–nucleic acid conjugates. In 1997, He became head of the Structural Chemo‐ and Bioinformatics Workgroup at the newly founded Max‐Planck‐Institute of Chemical Ecology, Jena, Germany.

In Autumn 2002, he moved to Cologne University Bioinformatics Center (CUBIC) as head of the Research Group for Molecular Informatics. In December 2003, he received his Habilitation in Organic Chemistry from Friedrich‐Schiller‐University, Jena, Germany. From 2008 to 2016, Christoph was head of cheminformatics and metabolism at the European Bioinformatics Institute (EBI) in Hinxton, Cambridge, UK. During this time, his group developed open chemistry databases for the biosciences, such as ChEBI, the dictionary and ontology of Chemical Entities of Biological Interest, and the MetaboLights database, a repository and reference database for Metabolomics.

At present, Christoph Steinbeck is Professor for Analytical Chemistry, Cheminformatics and Chemometrics at the Friedrich‐Schiller‐University, Jena, Germany. The Steinbeck group's research is dedicated to natural products research, the elucidation of metabolomes by means of computer‐assisted structure elucidation and other prediction methods, the reconstruction of metabolic networks, and algorithm development in cheminformatics. They further help developing a number of the leading open source software packages in chemo‐ and bioinformatics, including the Chemistry Development Kit (CDK), which was co‐founded by Christoph Steinbeck.

**Would you briefly explain what your research group is studying?**

The Steinbeck group's research is dedicated to natural products research, the elucidation of metabolomes by means of computer‐assisted structure elucidation and other prediction methods, the reconstruction of metabolic networks, and algorithm development in cheminformatics.

**Why did you choose a career in cheminformatics?**

I was already drawn to computers and programming in pre‐Internet times before I actually started studying at the University. When I had then completed my studies and started working as a researcher in the laboratory, I quickly realized that numerous tasks such as the structure elucidation of organic compounds could be automated. I then combined my passion for computer science and chemistry and started a career in cheminformatics. I never regretted this move and today I spend a lot of my time building infrastructure for chemical information.

**Of all your research projects, which one was your favorite and why?**

It is always one of my current research projects. At the moment, for example, we are looking at using deep neural networks (DNNs) and machine learning to solve big data problems in chemistry, and I am absolutely fascinated by the capabilities of these DNNs. I am convinced that they will fundamentally change the way in which we do science and also many other areas of our daily lives.

**What is your vision as editor on Analytical Science Advances?**

I am a strong advocate of open science, which requires open data and open access to information. I am excited that one of the established, high‐quality publishers is launching a full OA journal for the analytical sciences where we can start to (a) publish excellent science and (b) support the principles of open science, for example, by requiring the deposition of data which are underlying our articles in public repositories so that others can reuse the data and validate the findings in our articles.

**What do you think is the key to success in a scientific career?**

It is tempting to say “excellence,” but I think probably persistence is even more important. Scientists also need to have a certain resistance against frustration. Pursuing a vision over potentially decades requires quite a bit of persistence, in particular if you move against the mainstream. Good ideas will eventually succeed, but it is very understandable that not everyone is prepared to invest his or her lifetime to make that happen.

**Who were the most influential people in your career?**

I was exceptionally lucky to meet like‐minded people in all phases of my career, who helped me to pursue my vision for open science in chemistry. Before becoming an independent PI, I also had the privilege to work with a number of outstanding mentors who supported me even if I didn't listen to their advice.

**As a mentor and advisor, what do you advise your students in general?**

Over the years you start to detect patterns in the careers and projects of your students and co‐workers. PhD projects, for example, almost always follow the same path, from initial uncertainty, to the first successes, periods of enthusiasm, valleys of disappear, and eventually an extremely productive final period leading to good scientific results and a nice thesis.

Being able to convey this to your students in a friendly and caring way is already a great help for them. Furthermore, in our post‐factual time, it becomes more and more important to remind myself and my students, how important rigorous and honest scientific work is, and that there are no shortcuts to good science.

**What do you consider to be the more exciting topics in analytical chemistry?**

Again, at any point in time, I have always been excited by the latest discoveries in the analytical sciences. And of course, it is very much depending on your application scenario whether a certain topic is exciting or not. For me as a natural product chemist, nuclear magnetic resonance spectroscopy (NMR) was always an essential tool for structure determination and I am still fascinated by the power of 2D NMR for this purpose. This is also why, from the current set of new methods, cryo‐EM method microcrystal electron diffraction is exciting, and I am curious how generalizable this method will turn out to be for the structure elucidation of natural products.

**What are your views on the future of your field?**

Having observed science in general, and the analytical sciences in particular, over more than 30 years now, I'm convinced that the steady stream of stunning innovation that we have witnessed in the past, will not dry up.

**What are your favorite past times outside of science?**

There are too many to mention: Spending time with my wife and my cats, physical activities such as yoga, diving, making (wood working, electronics, 3D printing), but also leisure, good wine, good food. My day never has enough hours, also because I am not one of those lucky individuals who can live with 4 hr of sleep per day.

**What would you do if you had 1‐year paid leave?**

Fifty percent of it for extended diving trips with my wife around the world, the other 50% for making stuff in my workshop.

**What nonscientist inspires you the most?**

Currently, Elon Musk.

